# Mapping forest cover change and estimating carbon stock using satellite-derived vegetation indices in Alemsaga forest, Ethiopia

**DOI:** 10.1371/journal.pone.0310780

**Published:** 2025-02-06

**Authors:** Anbaw Tigabu, Agenagnew A. Gessesse

**Affiliations:** 1 Department of Geography and Environmental Studies, GIS and Remote Sensing, College of Social Sciences and Humanities, University of Gondar, Gondar, Ethiopia; 2 Department of Geography and Environmental Studies, College of Social Sciences and Humanities, Kabri Dahar University, Kabri Dahar, Ethiopia; 3 Department of Remote Sensing Research and Development, Space Science and Geospatial Institute, Addis Ababa, Ethiopia; Van Lang University: Truong Dai hoc Van Lang, VIET NAM

## Abstract

Deforestation and forest degradation are significant threats, leading to a decline in forest cover change, biomass and carbon storage, a crucial factor in mitigating climate change. Remote sensing techniques using satellite imagery offer a valuable tool for efficiently monitoring forest cover and biomass over different areas. This study aimed to map and quantify the forest cover change, biomass and carbon stored in the Alemsaga forest, Ethiopia. The study employed Landsat satellite images from four different periods (1992, 2003, 2013, and 2022) to track changes in forest cover and construct carbon storage maps for the Alemsaga forest. The findings from this study can be used to develop better forest conservation and management strategies. The study revealed a significant increase in dense forest cover in Alemsaga (35.34%) between 1992 and 2022, now encompassing 48.25% of the total forest area. Notably, satellite-derived vegetation indices (NDVI & DVI) exhibited a strong correlation with ground observations (R^2^ = 0.80), and statistical analysis confirmed this relation with above-ground carbon levels (R^2^ = 0.84). This enabled the creation of carbon storage maps, revealing a substantial increase from 159.31 t/ha in 1992 to 323.84 t/ha by 2022. It’s important to acknowledge that while NDVI/DVI proved effective, other factors might influence carbon storage. However, the study clearly shows that satellite imaging has the capacity to map forest cover change, biomass and estimating carbon stock accurately, which is an important first step toward a better understanding of how forests contribute to climate change.

## Introduction

All living things include carbon, which is essential for life on Earth [1, 2]. Above-ground biomass (AGB) is a significant carbon store in forests (70–90%) and an important indicator of forest health, reflecting its forest stages [3, 4]. Accurate carbon measurements can help guide forest management and development efforts to combat climate change by increasing carbon storage in soil and forest [5]. Field measurements provide the most reliable forest carbon data; but, they are expensive and time-consuming for wide areas [6, 7].

Remote sensing (RS) has recently emerged as a reliable and effective method of collecting data over wide areas, allowing for detailed forest mapping and tracking at both local and regional scales [6, 7]. RS, which detects how trees reflect light, can be used to map and track plant growth and biomass over time [8], as well as to collect information on forest cover change, biomass and carbon stocks [9]. Although it is not possible to quantify forest biomass directly, optical remote sensing is sensitive to variables that are correlated with biomass, such as tree size, density, and shadow (particularly in infrared wavelengths) [10]. Because of this, it is an affordable tool for biomass investigations, providing consistent data collection and a respectable level of accuracy at different forest scales [8, 11, 12]. Vegetation indices (VIs) are widely used in research to quantify biomass. VIs are used to interpret forest cover on land and are derived from satellite data [5].

Ethiopia is one of the most climate-vulnerable nations, and uncontrolled human actions and its effects on several sectors have recently become national issues [13, 14]. Thus, one of the areas of Ethiopia covered with forests is Alemsaga Forest. But starting in 1978, it was protected with the intention of restoring the deteriorated land, preserving the remaining natural forests, and serving as a seed source [13]. The region was transformed into farmlands and grasslands due to unlawful operations that occurred between 1990 and 1992, during the government transition. Following the country’s political stabilization in 1993, the study zone was once again designated as a forest [15].

While Alemsaga Forest has been studied before [15–17], this study combines the use of Geographic Information Systems (GIS) and satellite images (Landsat) to immediately produce a map of the forest’s carbon storage that corresponds with ground-based measurement references. Landsat’s free availability, wide coverage, and long history make it an important tool for forest assessments [7]. Therefore, this study used Landsat images from 1992 to 2022 to monitor forest cover change and estimate carbon storage in Alemsaga forest. The study developed models based on satellite-derived vegetation indices (VIs) and validated them with ground based measurements.

## Materials and methods

Researchers in this study used a combination of satellite data analysis (including preparing the data, classifying land cover, and extracting plant health indicators) with on-the-ground measurements. They then employed statistical methods to link changes in land use and land cover (LULC) to plant health (measured by vegetation indices), biomass and the amount of carbon stored (carbon stock). The findings were presented visually through maps and with statistical summaries.

### Study area description

**Fig 1:**
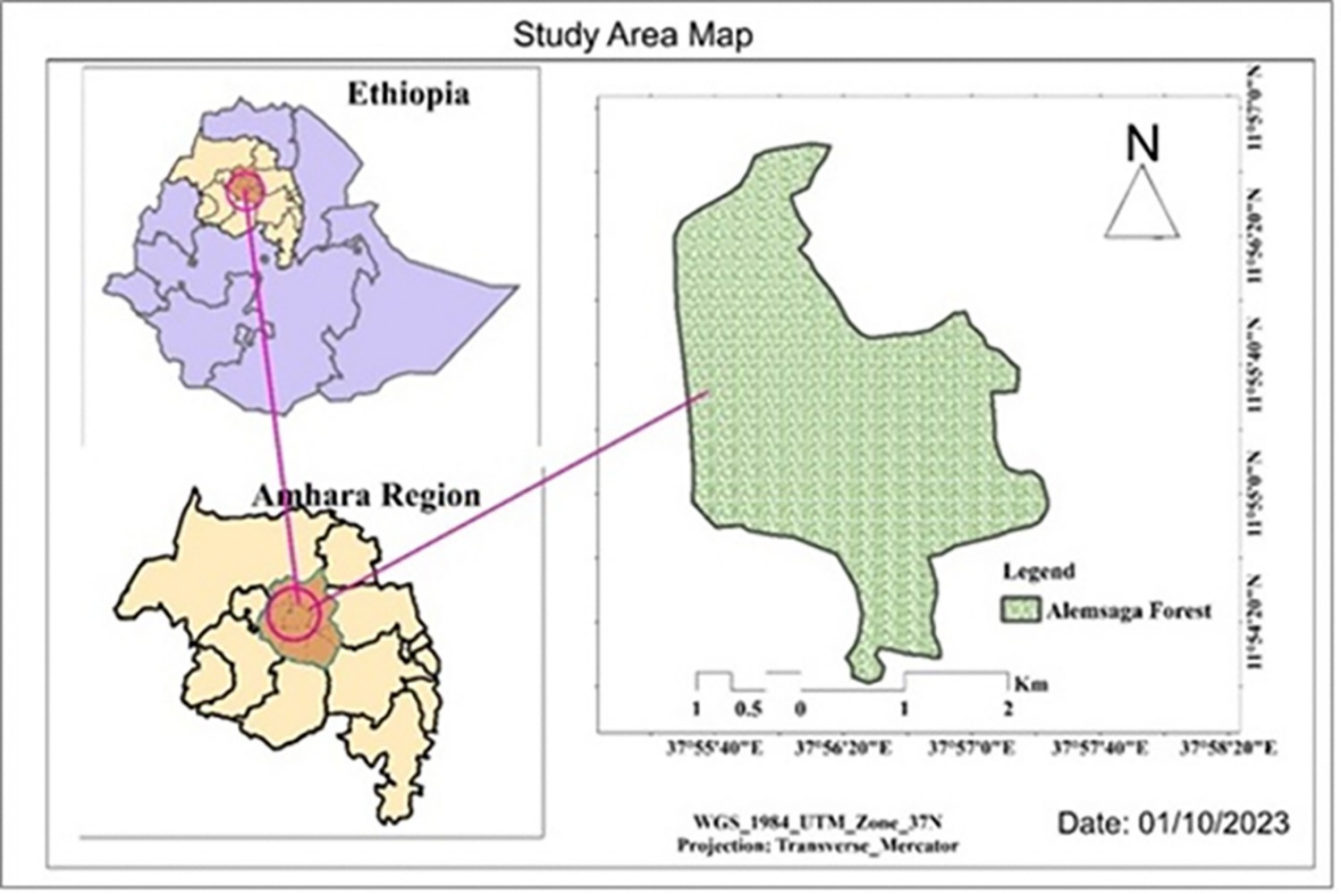
Location of the study area map.

The study was conducted on Alemesaga forest (here after called Alemsaga), South Gondar Zone, Amhara Region of Northwestern Ethiopia. Alemsaga is found between Farta woreda of Qoley, Dengors and, Arengoe kebele and Fogera woreda of Alember zuria kebele. It is located at 646 km north of Addis Ababa, 82 km east of Bahir Dar city and 20 km west of Debre Tabor town. The forest covers 1030 hectares (ha) including plantation around the edges of the forest and Fogera woreda forest conservation area [13]. From this 245 ha is covered by Fogera woreda and the remaining 785 ha is covered by Farta woreda South Gondar Zone Natural Resource Conservation Office. The forest is bordered in the north by Koley kebele, in the south Dengors, in the east Angore, and in the west by Alember Zuriya kebele. Geographically, the area is located at 11° 52’30"-11° 56’ 30" N & 37° 52’10"- 37° 58’0" E (Fig 1).

#### Bio-diversity of Alemsaga forest

Despite 44 years of protection since 1978, Alemsaga Forest faces challenges. Established to conserve remaining natural forests, provide seeds for plant restoration, and restore damaged areas, the forest struggles with disappearing tree species like Habesha tide and Woyra, while others like Berberta are used for local fishing practices [15]. So, Alemsaga has diverse plant species such as, Loonchisa (Bersama abyssinica), Anfare (Buddleja polystachya), lenquata (Grewiaferruginea), Warka (Ficusvasta), Kega (Rosa abyssinica), Kamo (Rhusglutinosa), Keret (Osyrisquadripartite), Woira (Oleaeuropaeasubsp Cuspidaten), Kitkita (Dodenaangustifolia), Agam (Carissaspinarum), Gumero (Capparistomentosa) Zegeta (Calpuri naaurea) Girar (Acacia abyssinica) and animal specious such as ape (Hominoidea), Monkey (Macaca mulatta), hayena (crocuta crocuta), Python (python molurus), tiger (panthera tigris), Blackbuck (Antelope cervicapra) are some of the animal species that are found in the forest [13, 16].

#### Climate and topography

**Fig 2:**
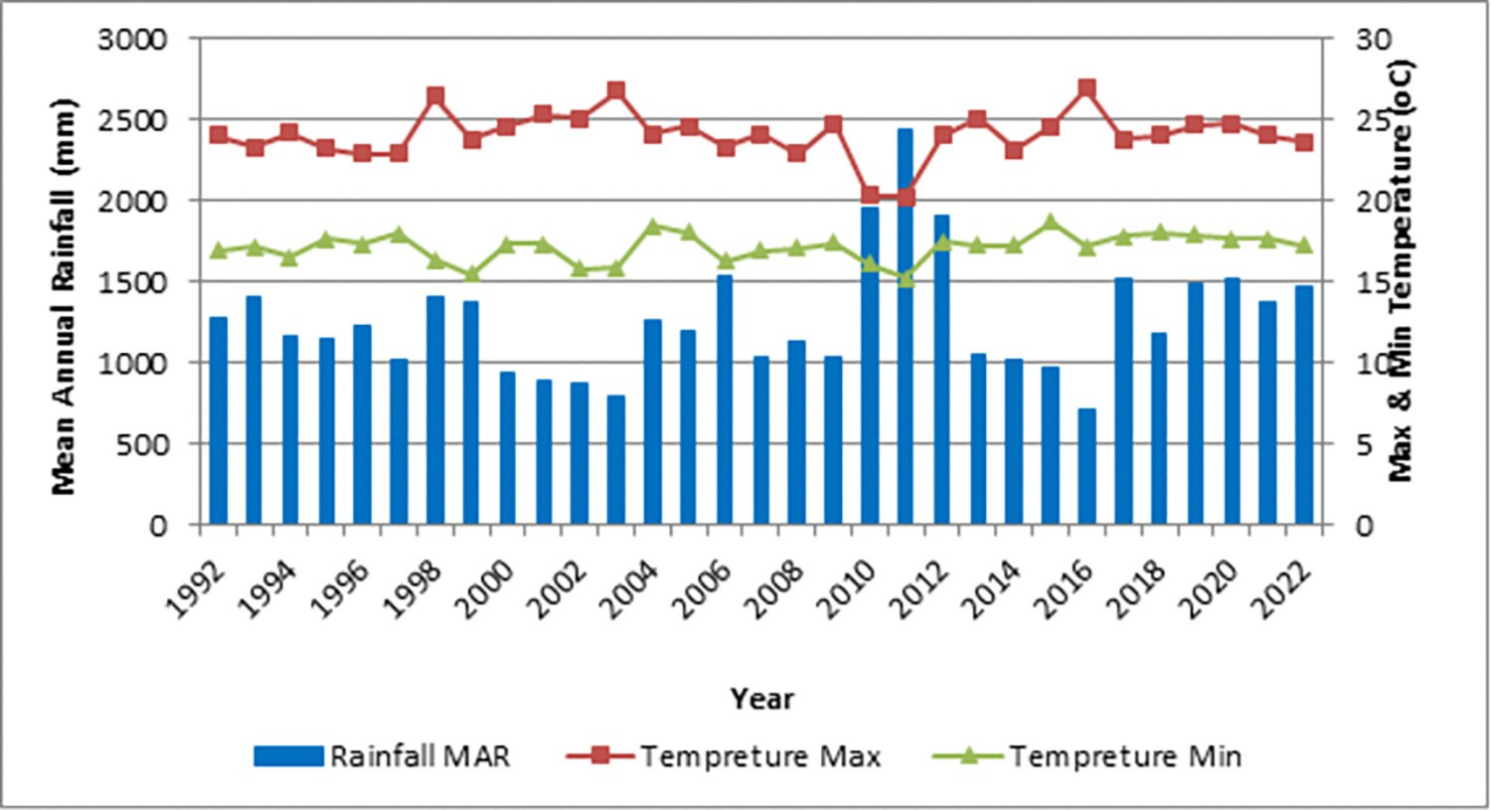
Temperatures and rainfall distribution of the study area.

The average annual minimum, maximum and mean temperature is 15.46°C, 26.49°C and 20.9°C respectively and the average annual rainfall of the study area is about 2486 mm and from this 85% of the rain falls during the wet season (June-September) (Fig 2).

A true driver of soil carbon cycling, the climate can influence soil carbon storage through both direct (e.g., emissions) and indirect (e.g., plant production and breakdown processes) effects on carbon cycling. High rates of plant productivity and litter breakdown are frequently observed in areas of Earth with high mean annual precipitation, ultimately resulting in significant soil organic production and atmospheric carbon-fixation rates. Conversely, areas with significant evapotranspiration, such those from dry land ecosystems, frequently result in decreased plant productivity, which eventually limits carbon-cycling and carbon storage. This is supported by the extensive body of literature which suggests that compared with medic environments arid regions have less plant biomass [18], leading to lower concentrations of soil carbon and nitrogen (biologically controlled) [19]. In terms of temperature, locations with high maximum temperatures should store less carbon dioxide (CO_2_) than those with lower temperatures. Hence, it is likely that the equilibrium between plant output and soil respiration was disrupted in areas with high maximum temperatures [20] is negative, suppressing carbon sequestration.

**Fig 3:**
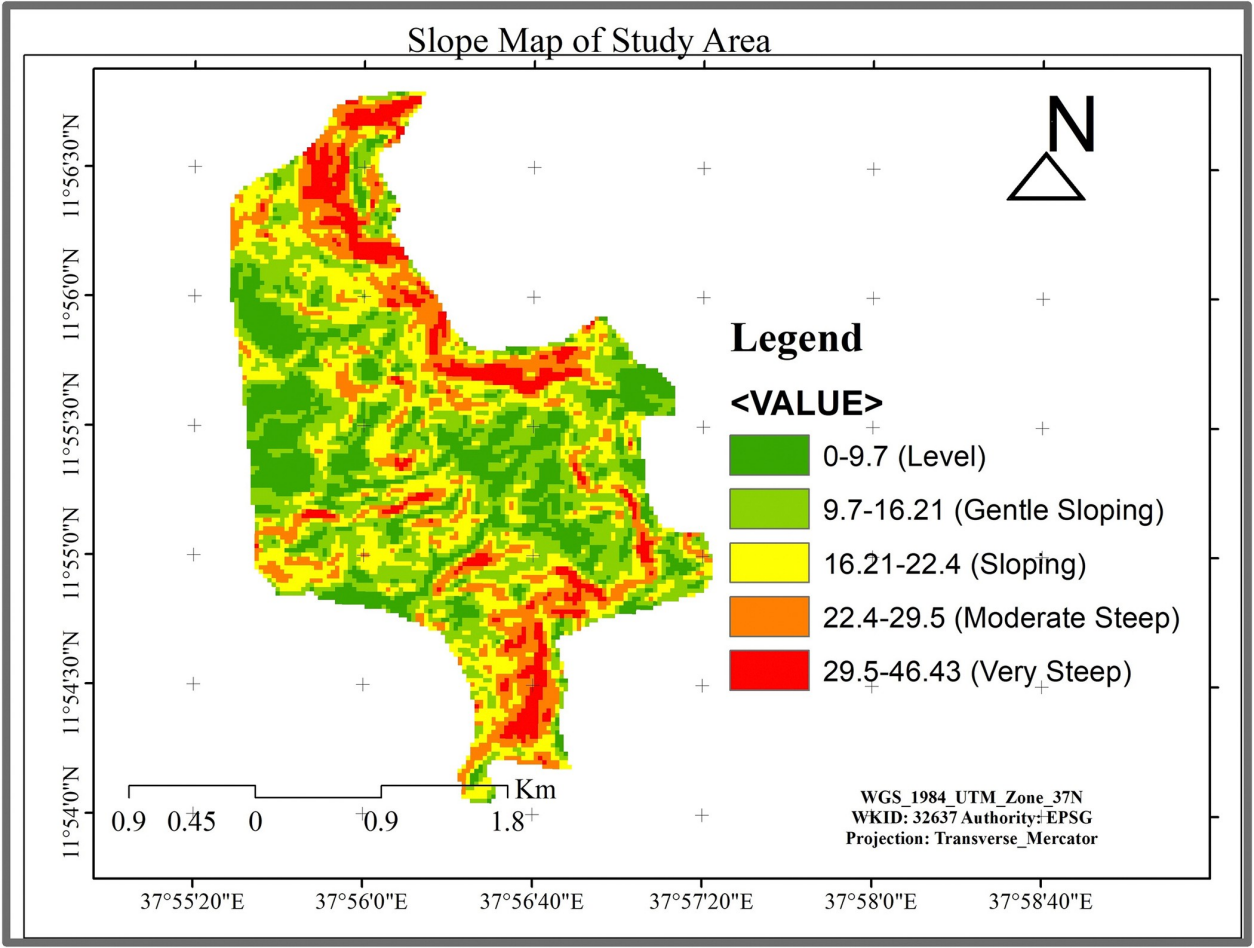
Slop map of Alemsaga forest based on FAO standard.

The topography of Alemsaga comprises uneven & rugged mountainous highlands, extensive plains & deep gorges (Fig 3). The altitude of the study area ranges from about 2079 m to 2550 m above mean seal level, and according to [21] research, topography of the area is high and steep mountain that influence the distribution of vegetation due to climatic condition (Fig 2). When the area is high mountain, it leads to low temperature and the area is plain medium slope, it leads to comfortable the distribution and growth of vegetation [22].

#### Data acquisition, pre-processing and software

The study was based on publicly available Landsat satellite imagery. These Landsat program images are available for free download via a number of websites within 24 hours of acquisition [23]. Specifically, four multi-spectral Landsat scenes with a resolution of 30 m were used in this study. The imagery was taken from the NASA-maintained public domain archive Earth Explorer, which is a data access facility provided by the United States Geological Survey (USGS) at https://earthexplorer.usgs.gov.

The four period Landsat image sensors on board Landsat-5: Thematic Mapper (L-5: TM) for 1992, Landsat-7: Enhanced Thematic Mapper Plus (L-7: ETM+) for 2003 & 2013 and Landsat-8: Operational Land Imager (L-8: OLI) were taken dry periods (January to March) of cloud free in the path/row is 169/052. The L-7 uses ETM+ sensor to capture images in seven different spectral bands with a spatial resolution of 30-m for Bands (B-1-5, and B-7). The spatial resolution for B-8 (panchromatic) is 15-m. L-8 equipped with instruments OLI and TIRS is capable of capturing images in nine different spectral bands with a spatial resolution of 30-m for B-1-7 and B-9 [21, 22]. Bands (B-2 to B-7) of multi-spectral L-8 images and B-1-5 and -7 of multi-spectral L-7 images were compiled to form the input band-set images for LULC and VIs categorization.

These individual band-sets serve as input image for training and classification. Additionally, the study employed field-collected GPS data and high-resolution Google Earth imagery as reference points to validate the Landsat image classification results. This verified the analysis’s accuracy by evaluating the model’s output with actual field observations. The preparation of Landsat images for analysis involved multiple stages. To correct for atmospheric interference and modify pixel values, they were first radiometrically corrected. Subsequently, the images underwent stacking and co-registration to guarantee precise positioning. Lastly, visual inspection using various band combinations assisted in determining the distribution of land cover.

Using both open-source and commercial tools, the study employed QGIS for raster visualization, ArcView for additional analysis, ERDAS Imagine for land cover data extraction and vegetation indices, and SPSS for statistical analysis (including regression modelling). In addition, the data was displayed using standard Microsoft tools.

### Image processing and LULC classification

Landsat’s optical multispectral images were analyzed to investigate spatiotemporal forest cover changes and related vegetation indices in Alemsaga, Ethiopia. This study utilized remote sensing data from Landsat satellites combined with GIS/QGIS to automatically analyse these changes. Spectral characteristics, the unique way different land covers reflect light, allow researchers to identify and categorize various elements on Earth’s surface [23, 24]. By examining these spectral signatures, the study can categorize different forms of land cover and monitor how forests have changed over time. The analysis was further refined using vegetation indices, which were derived from the spectral data and reflect the health of the vegetation. This was an enable us to compare changes in vegetation health as well as pre- and post-forest cover changes.

**Fig 4:**
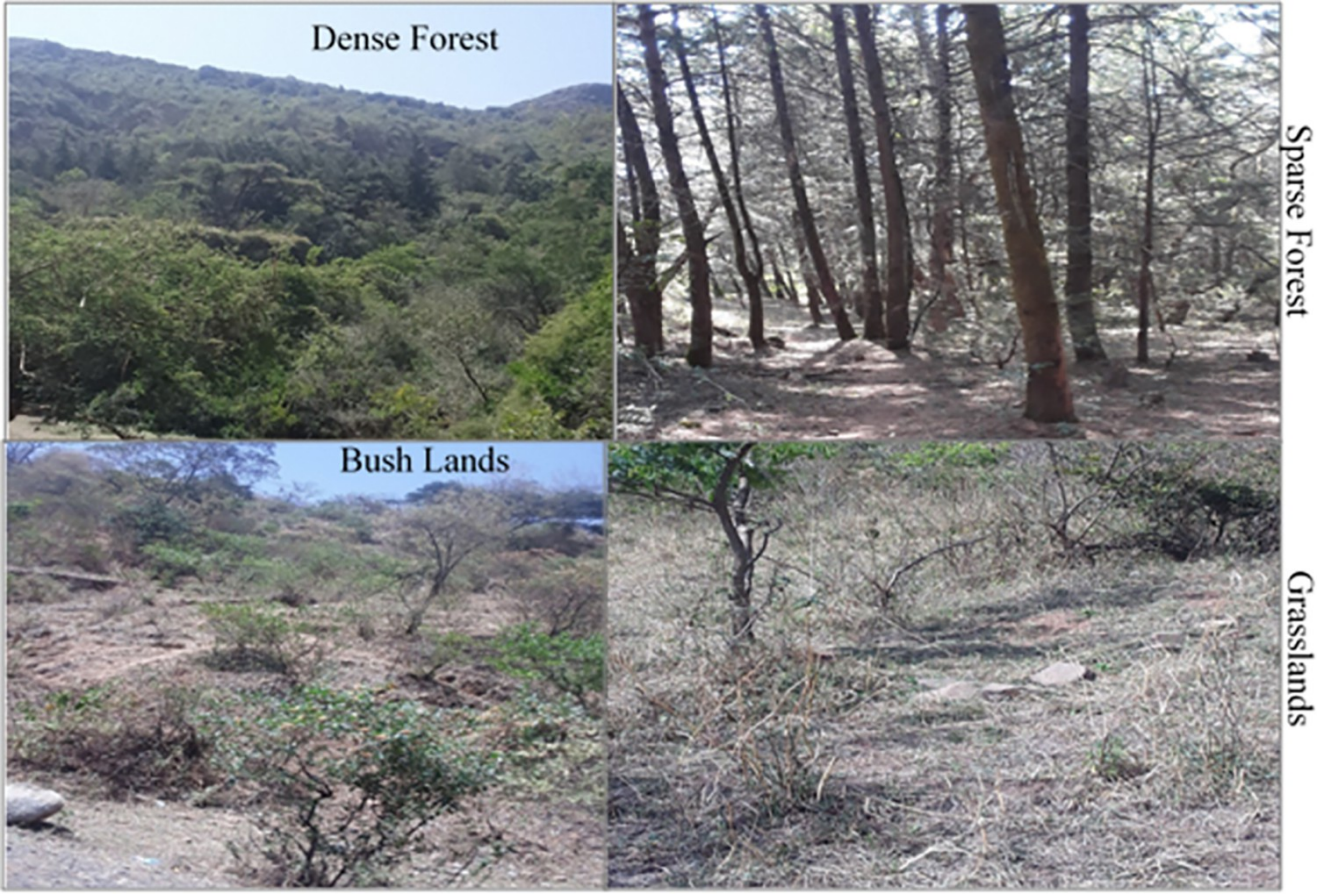
Land cover types distribution in the study area.

ERDAS Imagine 2015 was used to classify and interpret the Landsat images. To ensure accurate classification, all data were standardized to the same projection system (UTM Zone 37 N, WGS-84 datum). A supervised classification approach was employed, where training data sets (signatures) were created for each land cover class using over 150 reference points per Landsat image. These training points were carefully selected to represent each land cover type proportionally (with at least 20 points per class). Finally, the signatures were used to classify the images in ERDAS Imagine, resulting in a map depicting four main land cover types: dense forest, sparse forest, bush lands, and grasslands (Table 1 and Fig 4).

**Table 1:** Distribution of land cover types in the Alemsaga forest.

No	LULC Types	Descriptions
**1**	**Dense forest**	Areas covered with close growth of trees
**2**	**Sparse forest**	Area covered with scattered trees
**3**	**Bush lands**	These include shrubs and young tree species
**4**	**Grasslands**	Land covered by grass with scattered shrubs

#### Accuracy assessment

To ensure the reliability of the classified land cover map, an accuracy assessment was conducted in ERDAS Imagine 2015. This involved comparing the classification results with reference data representing "true world" land cover classes. That is, using an error matrix that calculated the classification’s kappa coefficients and overall accuracy, the classification results and true world classes were compared [25, 26]. In ERDAS Imagine 2015, an accuracy assessment was completed [27, 28].

### Deriving spectral indices

Ten spectral indices were calculated from the Landsat imagery and combined with the original bands to create a more diverse dataset for classification (details in Table 2). These indices, derived from mathematical combinations of spectral bands, are known to improve vegetation indices classification because they capture biophysical properties of the land surface [21, 29]. Spectral indices offer several advantages: they are unbiased, can be applied consistently across large areas (scalable), require minimal processing time (rapid), and provide quantitative information for analysis [24].

**Table 2:** Landsat data and input variables considered in regression analysis.

Role	Landsat-5 (TM)	Landsat-7 (ETM+)	Landsat-8 (OLI)
**Band name & description**	Band1: 0.45–0.52μm (Blue) Band2: 0.52–0.60 μm (Green) Band3: 0.63–0.69 μm (Red) Band4: 0.76–0.90 μm (NIR) Band5: 1.55–1.75μm (SWIR1) Band7: 2.08–2.35μm (SWIR2)	Band1: 0.45–0.52μm (Blue/Blue-green) Band2: 0.52–0.60 μm (Green) Band3: 0.63–0.69 μm (Red) Band4: 0.76–0.90 μm (NIR) Band5: 1.55–1.75μm (SWIR1/MI) Band7: 2.08–2.35 μm (SWIR2/MI)	Band2: 0.45–0.52μm (Blue) Band3: 0.52–0.60μm (Green) Band4: 0.63–0.68μm (Red) Band5: 0.85–0.89μm (NIR) Band6: 1.56–1.66μm (SWIR1) Band7: 2.10–2.30μm (SWIR2)
**Vegetation Indices**	**Variables and formula**		**References**
	*VI* = *NIR*−*R*		[30]
	$NDVI=\frac{NIR-R}{NIR+R}$		[31]
	$EVI=2.5\left(\frac{NIR-R}{NIR+(2.4*R(+1))}\right)$		[32]
	$GDVI=\frac{NIR-G}{NIR+G}$		[33]
	$SAVI=\frac{NIR-R}{NIR+R+L}(1+L)$		[32]
	$MSAVI=\frac{2*NIR+1-\sqrt{\left({2*NIR+1)}^{2}-8*(NIR-R\right)}}{2}$		[34]
	$OSAVI=\frac{NIR-R}{NIR+R+0.16}$		[35]
	$SARVI=\frac{NIR-R}{NIR+R+l}$		[30]
	$TNVI=\frac{NDVI+0.5}{NDVI+0.5}\sqrt{NDVI+05}$		[36]
	$MNDVI\;=\frac{\left(SWIRmax–SWIR\right)}{{SWIR}_{max}–{SWIR}_{min}}$		[36]

Several particular indices are mentioned in the text, one of which is the commonly used Normalized Difference Vegetation Index (NDVI) for vegetation cover characterization [25, 37]. Some, such as the Modified Normalized Difference Water Index (MNDWI), minimize interference from soil and plant while effectively extracting water features [34]. These additional indices DVI (difference vegetation index), GDVI (green difference vegetation index), SAVI (soil adjusted vegetation index), TNDVI (transformed normalized difference vegetation index), EVI (enhanced vegetation index), SAAVI (soil and atmospheric adjusted vegetation index), OSAVI (optimized soil adjusted vegetation index) and MSAVI (modified soil adjusted vegetation index) were also generated from a combination of different bands of Landsat [32, 38, 39]. They typically have straight forward algebraic formulations [21, 25, 40].

The study compared the effectiveness of various vegetation indices for mapping aboveground carbon (AGC) stock. These indices were categorized based on their ability to handle soil background influences:

- Common vegetation indices (DVI, NDVI, TNDVI, GDVI)
- Soil-adjusted indices (EVI, SAVI, OSAVI)
- Other vegetation indices (SARVI, MSAVI). Red (R), Green (G), and Near-Infrared (NIR) bands from the Landsat imagery were used to calculate these indices (see mathematical calculations in Table 2).

Here are some key points about the chosen indices: a) SAVI uses an L value of 0.5 to minimize the influence of soil background pixels, and b) NDVI values range from -1 to 1, with positive values indicating vegetation. Higher values (0.1 to 0.99) represent denser vegetation cover, while values close to -1 indicate bare land [25, 31, 37]. So, a stepwise linear multivariable regression analysis was performed to identify the relationship between AGC, the calculated vegetation indices, and the NIR band for the study area. Notably, the NDVI used in this analysis was derived from the R and NIR bands for the four years (1992, 2003, 2013, and 2022).

### Sample plots

**Fig 5:**
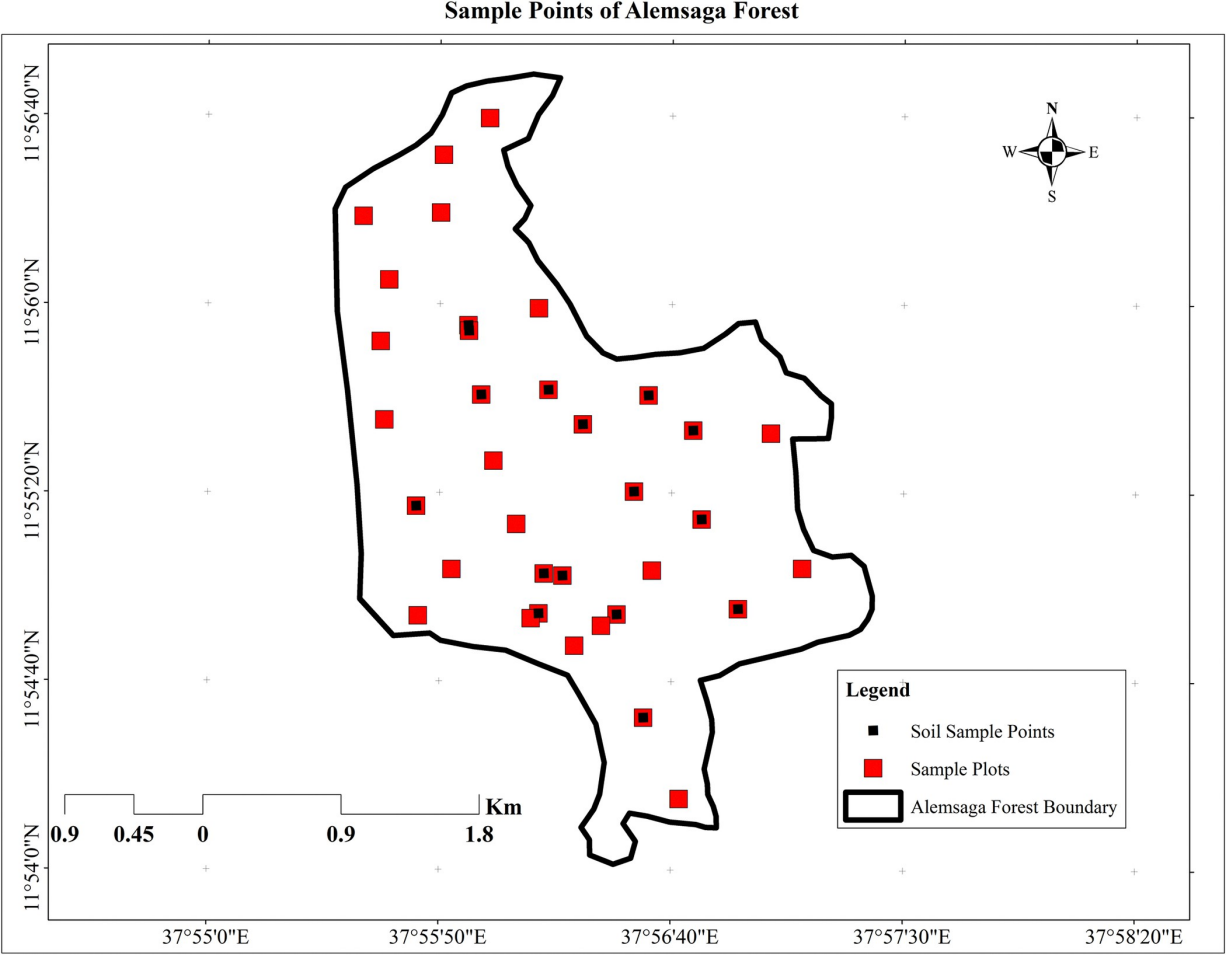
Sample plot map of the study.

To save time and money, the study employed a simple random sampling technique to select sample plots within the study area. This approach assumes relatively uniform climatic conditions and agro-ecological zones across the area. A total of 36 square-shaped sample plots, each measuring 16m x 16m (256 m^2^ or 0.00256 ha), were established for collecting both surface and subsurface data. This square plot shape is a common choice for estimating and mapping forest carbon stock or biomass across various vegetation types, including forests, plantations, agroforestry systems, shelterbelts, grasslands, and croplands. Square plots offer advantages in terms of ease of establishment, efficient identification of trees within the plot, and straightforward recording of GPS coordinates for relocation and monitoring purposes (as illustrated in Fig 5). A GPS device was used to locate each sample plot in the field.

**Fig 6:**
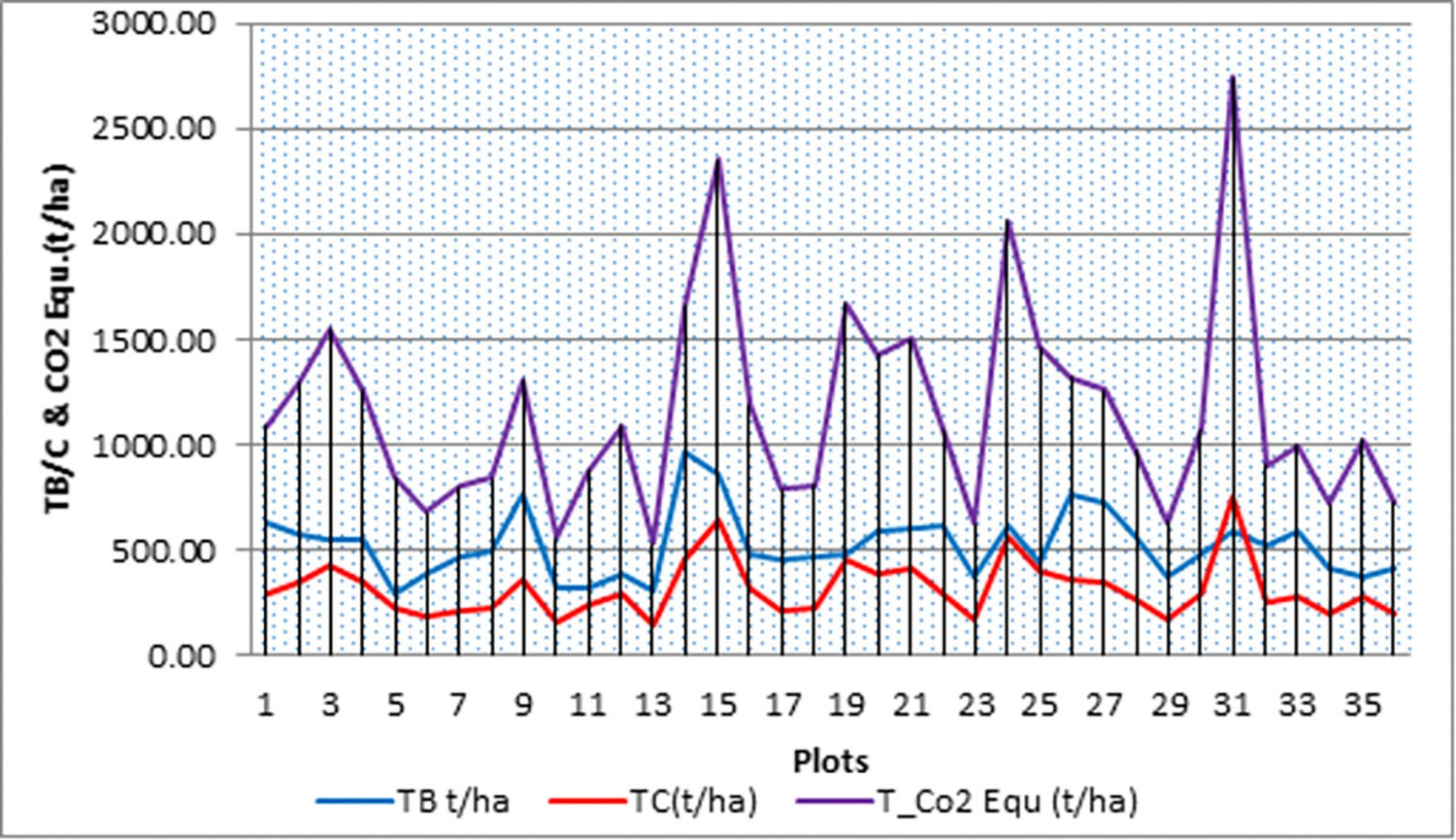
Mean distribution of total biomass & carbon stock value within the plot.

In Fig 6, the collected samples arrangement were done after the data collected, and then delivered for laboratory analysis to the Gondar Soil Testing and Fertility Management Centre (GTFMC). In soil laboratory room the experts who used soil dried water, core samplers and determination of soil organic carbon in percentage. According to the laboratory results, the sample soil’s mean organic carbon values were 1.99±0.82%, with plot 15 having the highest percentage of organic carbon at 4.04% and plot 14 having the lowest proportion at 0.93%. The study determined that the average amount of soil organic carbon (SOC) stored in Alemsaga forest ranged from 70.75 to 475.45 t/ha, with a mean of 159.59±104.63 t/ha.

The highest aboveground carbon stocks in plot 15 and the highest soil carbon stock values when compared to only 16 soil samples—not including the other aboveground carbon stock values—are thus correlated with soil carbon in this study (see Fig 6). As a result, collecting ground carbon stock sample requires on-site sampling.

### Statistical analysis

In the field, measurements of the carbon stock were taken using the ground. Furthermore, data were taken from satellite imagery of Landsat-5 (TM), Landsat-7 (ETM+), and Landsat-8 (OLI & TIRS). Correlation analysis and regression modeling were carried out using statistical analysis software (SPSS). Finding important field data variables that could be utilized to forecast aboveground carbon stock was the goal. This included: a) Field data variables with a strong positive association (high coefficient of determination, R^2^) with aboveground carbon stock were identified by correlation analysis. This shows that these variables account for a substantial portion of the variation in carbon stock; b) A model to predict aboveground carbon stock (dependent variable) was developed using regression analysis to identify variables and remote sensing data (independent variables); c) The model’s performance was evaluated using two key metrics: A low value for the Root Mean Square Error (RMSE) denotes a close fit between the model’s predictions and the actual field measurements. On the other hand, a high value (preferably near 1) for the Coefficient of Determination (R^2^) suggests that a significant amount of the data can be explained by the model [33, 35]. So, the validity of the regression models were evaluated using the RMSE and the R^2^ as follows:

$$R^{2}=1-\frac{\sum_{i=1}^{N}{\left(y_{i}-{\hat{y}}_{i}\right)}^{2}}{\sum_{i=1}^{N}{\left(y_{i}-\vec{y_{i}}\right)}^{2}},\;0\leq R^{2}\leq 1\;and$$ (1)

$$RMSE=\sqrt{\sum_{i=1}^{N}\frac{{\left(y_{i}-{\hat{y}}_{i}\right)}^{2}}{N}}$$ (2)

*y*_*i*_ is the observed value of carbon, ${\hat{y}}_{i}$ is the carbon calculated, and $\vec{y_{i}}$ is the predicted carbon by model of the response variables, and N is the number of observations.

Finally, this study relied on field data collection to validate the model’s accuracy in estimating carbon stock using satellite imagery. Field measurements included Diameter at Breast Height (DBH), tree Height (H), Aboveground Biomass Carbon (AGB/C), Belowground Biomass Carbon (BGB/C), Dead Wood Biomass Carbon (DWB/C), and Soil Organic Carbon (SOC) [39]. Statistical analysis of this field data played a crucial role in model validation [41], and it is calculated as the square root of the variance:

$$S=\sqrt{\frac{1}{N-1}\sum_{i}^{N}{\left(y_{i}-\bar{y}\right)}^{2}}$$ (3)

Where, S is the sample standard deviation, *y*_*i*_ is the sampled value, N is the number of samples (i = 1, 2… N), and $\bar{y}$ is the average value in the given samples.

### Allometric equations

Allometric equations are essential tools for estimating carbon storage in forests [42, 43]. These equations establish mathematical relationships between easily measured tree features, like diameter at breast height (DBH), and more challenging measurements like total biomass or carbon stock store [44, 45]. In essence, allometric equations act as a conversion factor, allowing researchers to convert readily available data (e.g., DBH measurements from forest inventories) into estimates of total biomass or carbon stock. This is particularly valuable in tropical forests with high tree diversity, where measuring every tree’s biomass directly would be impractical [42]. Here, allometric equations can be developed for specific forest types or ecological zones to provide reliable carbon estimates using just DBH measurements [36, 41, 44].

## Results and discussion

### LULC classification and accuracy assessment

Over the past three decades, a common approach for analysing land cover changes has involved using quantitative methods for LULC classification [46]. This essentially creates maps of land cover types. Then, by comparing corresponding pixels in these maps from different time periods, the study can identify and quantify the areas that have undergone change. First, the LULC classification results and related accuracies were demonstrated [47]. Second, the spatiotemporal change of the land use dynamics (the area (A) can be expressed in hectare (ha) and percent (%)) in the study area were shown (Table 3). Overall accuracy and kappa coefficient of image classification were 80% & 0.73, 81% & 0.75, 84% & 0.79, 88% & 0.84 for the year 1992, 2003, 2013 and 2022, respectively.

**Table 3:** LULC 1992–2022 Statistical Information.

LULC Types	1992		2003		2013		2022	
	A (ha)	%	A (ha)	%	A (ha)	%	A (ha)	%
**Dense forest**	111.51	12.56	356.58	40.15	380.34	42.83	428.05	48.25
**Sparse forest**	309.69	34.87	163.53	18.42	111.15	12.52	225.63	25.43
**Bush lands**	203.31	22.89	246.51	27.76	228.6	25.74	139.28	15.69
**Grasslands**	263.52	29.68	121.41	13.67	167.94	18.91	94.274	10.63
**Total**	888.03	100	888.03	100	888.03	100	888.03	100

In 1992, an analysis of land cover revealed that sparse forest was the dominant vegetation type, covering over 309.69 hectares (34.87%) of the study area. Grasslands followed in extent, at nearly 263.53 hectares (29.68%). Bush lands occupied a smaller area of roughly 203.31 hectares (22.89%), while dense forest was the least common cover type, at only 111 hectares (12.56%) (Table 3). Sparse forest dominated the central region, while bush lands were clustered in the northwest, north, and east. Dense forest appeared in patches across central, eastern, and northeastern areas. However, the limited extent of dense forest compared to other land cover types suggests significant deforestation for settlements, grazing, and farming activities at that time.

**Fig 7:**
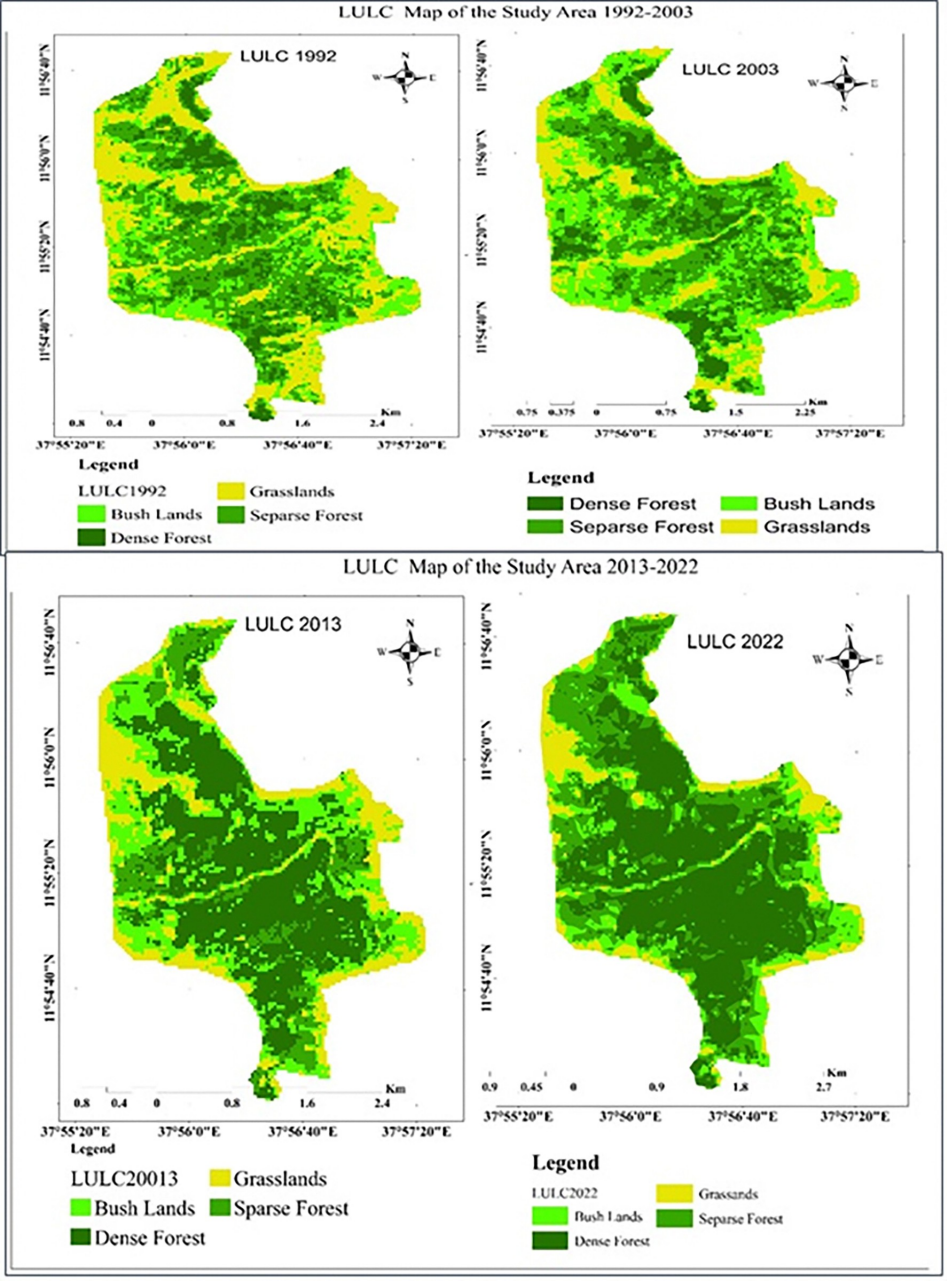
LULC map of Alemsaga forest for the years 1992, 2003, 2013 and 2022.

Land cover data from 2003 (Table 3) indicates a notable increase in dense forest cover 356.58 ha (40.15%) compared to 1992. This suggests potential improvements in forest protection. Bush lands (246.51 ha, 27.76%) remained the second most prevalent cover type. Sparse forest (18.42%) and grasslands (13.67%) showed a decrease in area, possibly due to the expansion of forested areas. It is implied in the text that conservation efforts may have made it easier for grasslands to change into various types of forests and shrub lands (Fig 7).

The LULC for 2013 (Fig 4) revealed that dense forests and bush lands covered the majority of the research region were 42.83% and 25.74%, respectively. During this time, spare forests made up the fourth land use cover, accounting for roughly 12.52%. This represents a loss of more than 5% from the preceding period. Here, grasslands make up the third land use cover (18.91%), however their share has grown significantly from 13.67 to 18.91%. As was already established, by enclosing the sparse forests, the dense forests were widely spread throughout the entire region. Grasslands are found on the peripheral or surrounding the forest region.

Similarly, as Fig 7 illustrates, in 2022, dense forest covered about 428.05 hectares (48.25%) of the total area. Sparse forest covered 225.63 ha (25.43%) of the entire area, up from 111.51 ha (12.56%) previously. Bush lands followed at 139.82 ha (16.69%) and grassland at 94.27 ha (10.63%). Hence, dense forest had the greatest area coverage in this year (2022) while grasslands had the smallest, indicating a decline in grasslands and an increase in dense forest from the start to the end of the period (1992–2022).

Finally, Fig 7 showed that all of the study area’s periphery is covered with grasslands, while the study area’s centre is entirely covered with dense forest. The middle section is covered with a variety of tree species and is unaffected by external attackers (like humans and livestock), despite the periphery being covered in grassland due to external attackers that affect the tree species.

### LULC change detection (1992–2022)

It is possible to identify real changes by directly comparing categorized images obtained on two different dates. The temporal changes that have occurred between two dates can be measured using a change matrix [47]. In this study, three periods of change detection spanning 30 years have been made: the first from 1992 to 2003, the second from 2003 to 2013, the third from 2013 to 2022, and the fourth from 1992 to 2022. This is very indicative of a lengthy history of LULC. Using the post-classification cross-tabulation method in ArcGIS software, the LULC change detection was evaluated [43, 48].

The area of grasslands and sparse forests changed by -142.11 ha (-16%) and -146.16 ha (-16.46%) between 1992 and 2003, respectively. Dense forest cover increased from 111.51ha (12.56%) in 1992 to 356.58ha (40.15%) in 2003. Conversely, bush lands had a change rate of 4.86% (43.2 ha), which is quite low. Between 1992 and 2003, there was a 245 hectare (27.6%) increase in the net dense forests. The areas of sparse forests decreased and changed into dense forests as a result of the protected and conserved forest area.

Between 1992 and 2003, there was a -146.16 ha (-16.46%) and -142.11 ha (-16%) change in the area of spare forests and grasslands, respectively. From 111.51ha (12.56%) in 1992 to 356.58ha (40.15%) in 2003, the area covered by dense forests grew. On the other hand, bush lands showed very little change, with a change rate of 4.86% (43.2 ha). The net dense forests grew by 245 hectares (27.6%) between 1992 and 2003. Because of the protected and conserved forest area, which transformed into dense forests, the regions of sparse forests reduced and became dense forests. The areas of sparse forests decreased and changed into dense forests as a result of the protected and conserved forest area. Over the same period of time, the amount of bush land increased at a pace of 43.2 ha (+4.86%) due to the conversion of grassland into bush land, which was covered in tiny trees and occasional bushes.

The net change in dense forest areas between 2003 and 2013 was only 23.76 ha (2.68%), which is little compared to the change in the rate of changes between 1992 and 2003. The area of grasslands expanded at a rate of +46.53 (5.24%) hectares each year between 2003 and 2013. There were decreased of -17.91 ha (2%), and -52.38 ha (5.90%) in bush lands and sparse forests. Comparably, the dense forests expanded by 428.05 ha (48.25%) of the total forest area in 2022, with a net change of 47.71 ha (5.37%) from 2013 to 2022.

The net change in dense forest areas between 2003 and 2013 was only 23.76 ha (2.68%), which is little compared to the change in the rate of changes between 1992 and 2003. The area of grasslands expanded at a rate of +46.53 ha (5.24%) each year between 2003 and 2013. Decreases of -17.91 ha (2%), and -52.38 ha (5.90%) were seen in bush lands and sparse forests. Comparably, the dense forests expanded by 428.05 ha (48.25%) of the total forest area in 2022, with a net change of 47.71 ha (5.37%) from 2013 to 2022. The area covered by scanty woods expanded by 225.63 ha (25.43%) at a rate of 114.48 ha (12.89%) over the same time period. Between 2013 and 2022, the remaining grasslands and bush regions, which made up -89.32 ha (10.06%) and -73.66 ha (8.30%) of the forest area, had a decline in covering area and changes in classification into dense and sparse forests, respectively. It’s true that areas with both sparse and dense forest cover sequester a large amount of carbon stock in comparison to other land use patterns. Because it employs photosynthesis to produce food and blocks the sun’s rays, which lowers CO_2_ emissions. Therefore, the forest region had a favourable climatic and stable ecosystem during the field observation period compared to the neighbouring habitats.

Overall, the LULC change analysis of the investigated regions indicated that the forest density is increasing at a rate of 316.54 ha (35.34%). However, the remaining LULC classes showed negative changes across the 30-year study area periods from 1992 to 2022. For sparse forests, shrub regions, and grasslands, respectively, the rate of change was -84.06 ha (9.47), -64.027 ha (7.21%), and -169.25 ha (19.06%) as a result of these changes.

### Vegetation indices for carbon stock value map of 2022

A vegetation index (VI), according to Huete et al. [32], is a spectral transformation of two or more bands that aims to enhance the contribution of vegetation features and allow precise comparisons of changes in terrestrial photosynthetic activity and canopy structural patterns over time and space. There are numerous variations of VIs, many of which share similar functional characteristics. Several of the indexes make use of the inverse relationship between red and near-infrared reflectance that is linked to healthy green vegetation. The primary goal of remote sensing is to characterize the kind, quantity, and state of vegetation that is present in the area.

**Fig 8:**
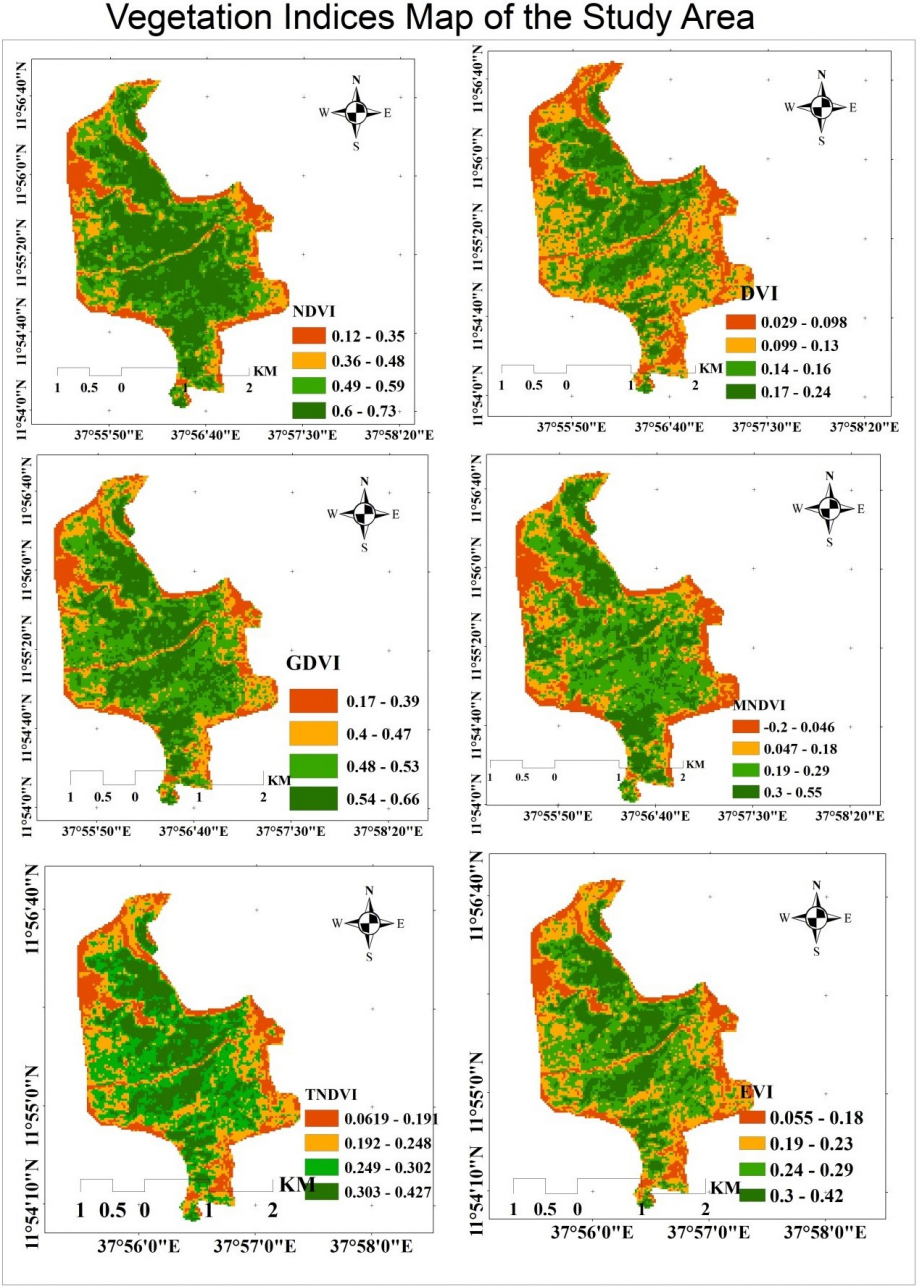
Vegetation indices map of the study area in 2022.

**Fig 9:**
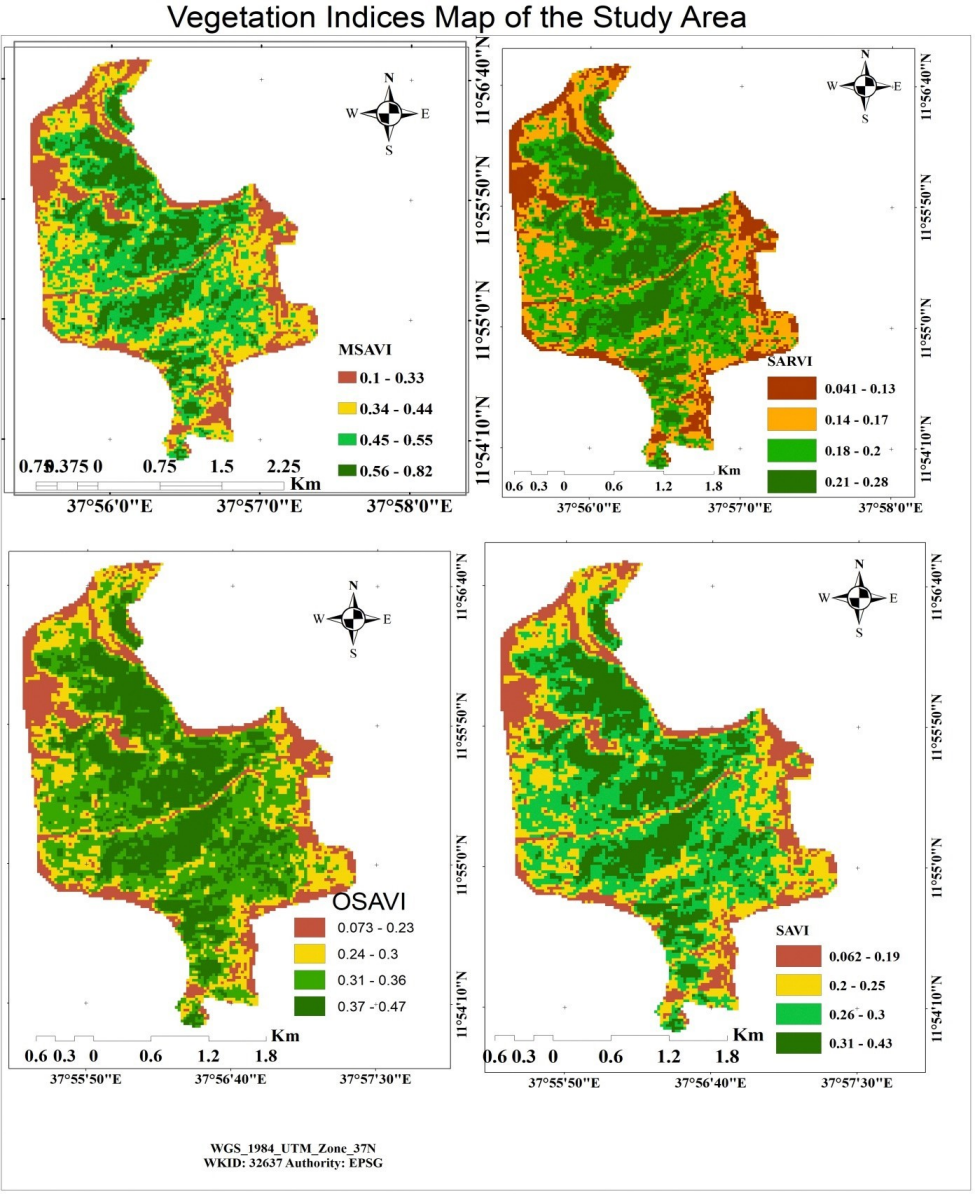
Vegetation indices map of the study area in 2022.

A VI can be computed by creating linear combinations of spectral band data and ratio differences and sums. In all, ten maps were made to show the several indices in the Alemsaga forest (Figs 8 and 9). The reflectance value and the carbon stock in the Alemsaga forest were estimated to be correlated via GPS coordinates.

Figs 8 and 9 showed that the study area’s centre had the highest NDVI forest density, while the study site’s periphery had the lowest value. For vegetation mapping and carbon assessment, NDVI is really one of the most widely used indices. The central part of the NDVI value was 0.73, whereas the outer or grassland area had the lowest value, representing stress. Similar to the NDVI and DVI, the EVI and GDVI were found in the centre of the forest area, with the north-eastern and eastern regions of the research having the lowest values. Due to the fact that the study area’s periphery was covered in grasslands and shrub lands, while the study area’s centre was heavily covered in a variety of plants types as well as dense forest. Similarly, the study created and extracted from Landsat images the SAVI, OSAVI, MSAVI, MNDVI, SARVI, and TNDVI maps. Based on the values of those indices, the study area’s periphery had the lowest vegetation index values, while the central region had the highest values. The estimates of the available biomass and carbon stock were significantly influenced by such vegetation indexes. Because there was a direct correlation between the parameters of the vegetation index and biomass, the indices values increased when carbon stock and biomass values increased, and vice versa. With a rating of 0.82, the MSAVI had the greatest vegetation index, while the EVI had the lowest, at 0.24.

### Correlation between vegetation indices & carbon stocks

The study employed a combination of data from field surveys and Landsat imagery to analyse the relationship and develop equations for the calculation of above-ground carbon stock (AGCS). The relation between the VI value obtained from Landsat data and the AGCS value was investigated using Pearson’s correlation. It calculates the degree to which two variables have a linear relationship. There was a high correlation between them if the coefficient was near to 1 [49]. Using the identification tool, the pixel values for every sample were retrieved. Regression correlation analysis was used to describe the correlation strength, pattern, and equation model of each parameter once the image values for these samples were collected. The correlation pattern in this study was found using as linear regression models as possible. The models that yielded the best prediction model were those whose "good fit" went through the majority of the depicted observation points [50]. The strength and type of the model between the tree carbon stock parameters and VI can be shown in the regression correlation analysis result. The dependent variable (y) was determined using the equation that was derived from the type of regression model [49, 51].

**Fig 10:**
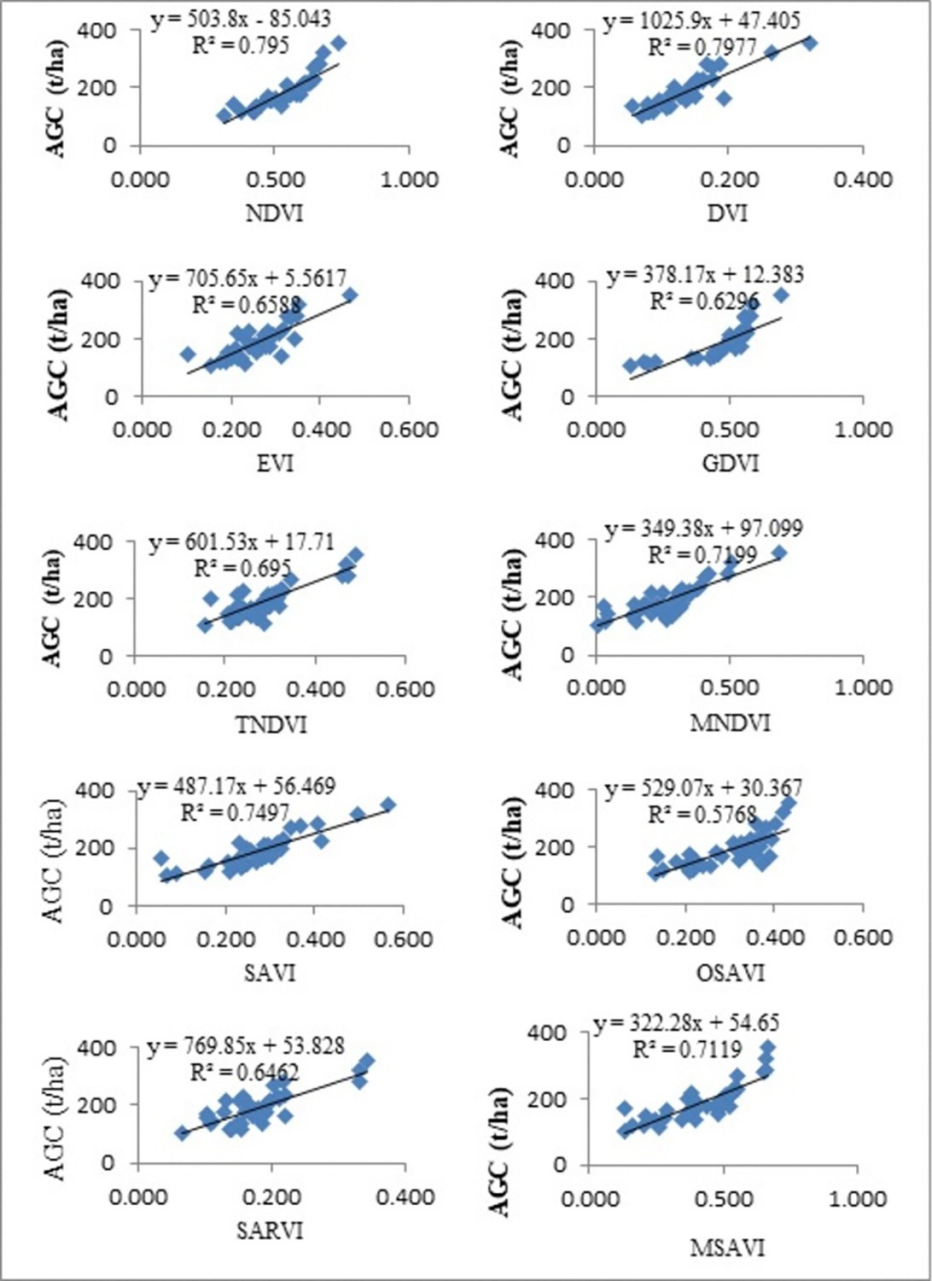
Scatter plots relationship between vegetation indices and AGC.

As shown in the Fig 10, the scatterplot between AGB values from field data and NDVI values derived from the Landsat-8 image the coefficient of correlation was 0.795, which means that ≈80% of the field data have been explained by the NDVI-based model using Landsat images. Because the values of R^2^ are close to 1 and greater than 0.5, then the correlation of AGB and NDVI values is strongly correlated between them.

In comparison among VIs, the correlation of NDVI values was greater than the rest of other indices (SARVI, MSAVI, OSAVI, SAVI, EVI, GDVI, MNDVI and TNDVI) except DVI. So, similarly, the correlation of coefficient between DVI and AGB values has been shown in the Fig 10 was 0.798 or ≈80% which has performed better as compared as NDVI with a strong relation. The rest of VIs correlation coefficient or regression model were calculated as follows. The values of EVI, GDVI, TNDVI, and MNDVI were calculated 0.69 (69%), 0.63 (63%), 0.695 (≈70%) and 0.72 (72%) respectively. Finally, the correlation between AGB values from field data and SAVI, OSAVI, SARVI, and MSAVI values derived from Landsat-8 images were 0.75 (75%), 0.579 (57.9), 0.65 (65%), and 0.71 (71%), while 25%, 42.1%, 35%, and 29% of the values were not explained by those variables, respectively. The SAVI value was the highest value and had the strongest positive correlation between AGB and the rest, whereas the OSAVI value had the lowest and most moderate correlation with AGB.

In general, the coefficient values of the VIs had high positive values; there were high vegetation covers, but the values of the vegetation indices were negative or zero, meaning there were no vegetation covers. This study showed that on the map, the central parts of all vegetation indices had the highest vegetation cover and the highest values of coefficient determination and regression of AGB/AGC with the vegetation indices.

### Model developed on predicted variables and AGB (2022)

Regression analysis was conducted because multi-collinearity and the variance inflation factor (VIF) value were used to check the study. Considering that the VIF serves as a gauge for the degree of multicollinearity among a group of multiple regression variables [52]. Regression analysis was not possible with the VIF and multi-collinearity tolerance values, which were less than 10 and greater than 0.1, respectively. The independent values of the residuals are another justification for using the regression model [22]. Durbin-Watson statistics was employed in this investigation to determine whether the residuals are independent. Durbin Watson values range from 0 to 4. The optimum regression is achieved if the value is near 2, however the regression is invalid if the value is less than 1 or more than 3. It is accurate to say that the Durbin-Watson result in this investigation was 1.83; it neared 2 and satisfied this premise. Regression analysis was employed in the study to correlate the independent values’ values [53, 54]. Furthermore, the statistical values for Cook’s distance were less than one, indicating the significance and application of the regression model [50].

The study built a linear regression model to link vegetation indices (VIs) from Landsat-8 satellite images with AGB/AGC stock data. Table 4 summarizes the best model identified from a comparison of multiple vegetation indices. These indices (VIs) showed strong positive relationships with AGB/AGC stock, with R^2^ values ranging from 0.57 to 0.79. Among the VIs tested, DVI and NDVI performed nearly equally well in predicting AGB/AGC stock, followed by MNDVI, another NDVI variant, SAVI, and MSAVI (see Table 4).

**Table 4:** Regression statistical summary of the AGB/AGC model.

No	Vegetation Indices	R	R^2^
1	**NDVI**	0.93	0.79
2	**DVI**	0.92	0.79
3	**GDVI**	0.80	0.66
4	**EVI**	0.78	0.63
5	**TNDVI**	0.83	0.69
6	**MNDVI**	0.86	0.72
7	**SAVI**	0.86	0.73
8	**OSAVI**	0.76	0.58
9	**SARVI**	0.79	0.65
10	**MSAVI**	0.85	0.71

The study developed multiple regression models in 2022 to estimate AGB/AGC stock using vegetation indices (VIs) derived from Landsat-8 satellite imagery. Among the ten VIs considered, DVI and NDVI were identified as the most effective independent variables for predicting AGC stock at a pixel level. A stepwise linear regression approach was likely used to select these optimal VIs. The specific equations used for this pixel-level estimation can be expressed as follows:

AGB=(584.322*DVI)+(283.846*NDVI)−46.406 (4)

AGC=(584.322*DVI)+(283.846*NDVI)−46.406*0.47 (5)

Analysis of the models (see Table 4) revealed that DVI and NDVI achieved the highest R^2^ values, both around 0.79. These were identified as the best performing models out of the ten vegetation indices tested. The equations for estimating AGC stock at a pixel level were likely developed using the coefficients (B values) of these chosen VIs and a constant term (beta value). A positive beta value would be added to the equation, while a negative one would be subtracted.

### Mapping of AGB & AGC stock (1992, 2003, 2013 & 2022)

**Fig 11:**
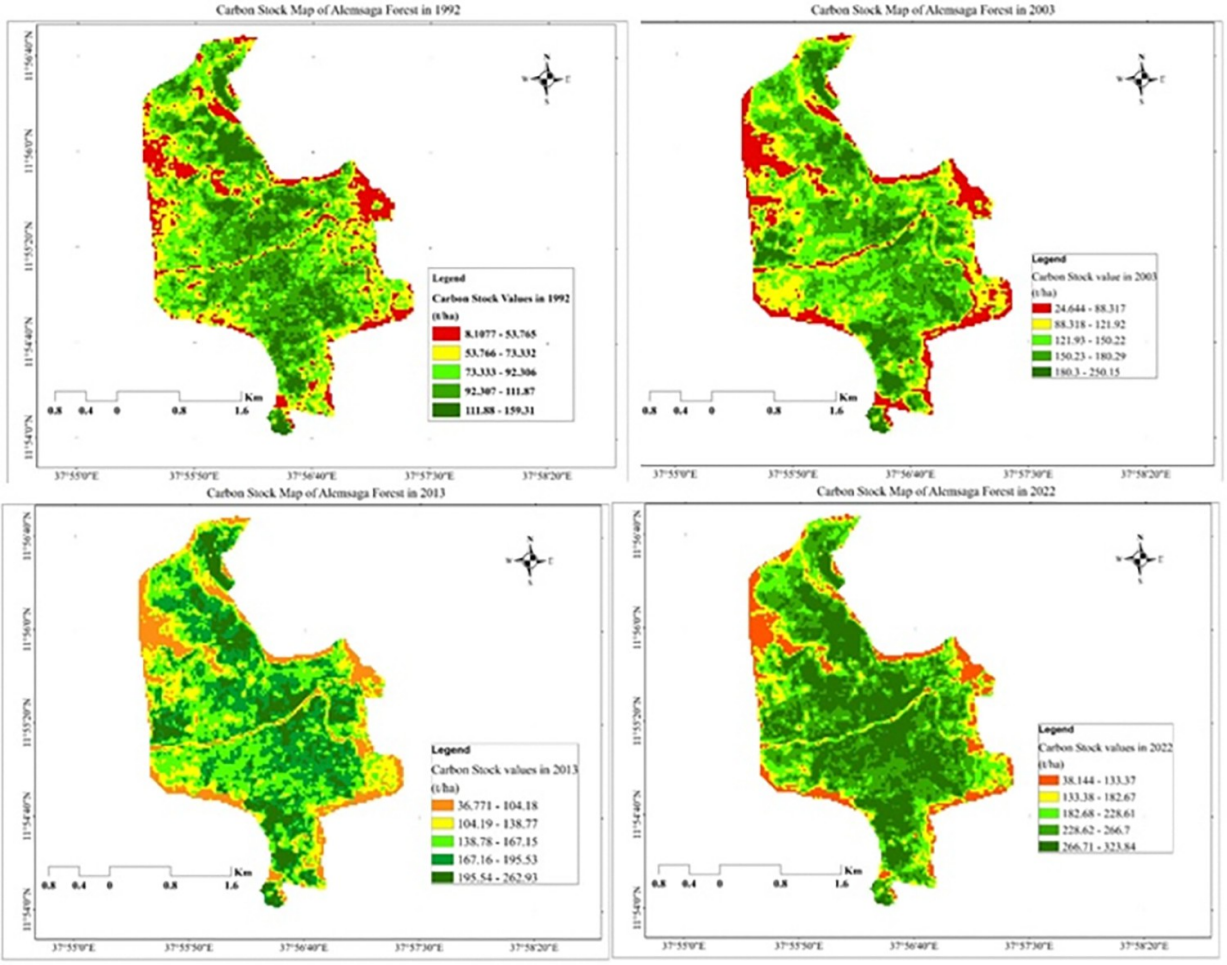
Carbon stock map of Alemsaga forest in 1992, 2003, 2013 and 2022.

According to [55], the distribution of the total AGC maps were created by the developed regression model of the interaction of the aboveground field data observation and VIs to develop the equations based on the best fitted and highest R^2^ values in 1992, 2003, 2013 and 2022 (Fig 10). The highest R^2^ was almost equal as 0.79 values and the lowest RMSE (root mean square error) was 0.086 or 8.6% of level of confidence was best for carbon map developed for DVI and NDVI indices. The equation was calculated on ArcGIS software using a raster calculator to produce the final AGC map (Fig 11).

In 1992, Fig 11 showed that the AGC stock was highly distributed in the central, northeastern, and southern parts of the study area, which covered 92.307–159.31 t/ha. Whereas in the northwestern, eastern, and all the peripheral parts of the study area, medium to low carbon stock values covered 8.1077–73.33 t/ha. The sum of the carbon stock values in the year 1992 was 834560 tone in all area coverage, and the average values were 84.58124.625 t/ha or t/pixel values because the carbon stock map was developed by using VIs values. Due to this reason, the study used t/pixel as the unit of measurement of AGC stock values in forest ecosystems. The same is true as observed in the field data collection time, the peripheral parts of the Alemsaga forest had low carbon stock values. Because the peripheral parts of the forest are affected by the surrounding community, including the animals, the trees were cut for the purpose of fuel, charcoal, house building, and grazing by their animals. In fact, grazing and farm lands have lower carbon stock than shrub and forest lands.

Similarly, in 2003, the minimum and maximum values of carbon stock value were 24.644 t/ha and 250.15 t/ha, respectively, which covered in the central and border parts of the study area. From the map, it is possible to see that the central, southern, and northern parts of the forest have the highest carbon stock values, whereas the western, south-western, eastern, and peripheral parts of the forest have the lowest carbon stock values. As compared, the amount of AGC stock values was higher than 1992. Due to the fact that the area is protected from external enemies, the coverage of forest by different tree species increased, which also increased the distribution of carbon stock over the sparse and dense forest. In 2013, the minimum, maximum, average value and sum of AGC stock values were 36.771t/ha, 262.93 t/ha, 155.27±38.484 t/ha and 1532000 ton, respectively. The result indicated that 12.78 t/ha of carbon stock values were increased from 2003 up to 2013 in the study area. Grass and bush lands are lower carbon stock values as compared from spares and dense forest land cover class in the Alemsaga forest land use land cover.

Finally, the spatial distribution of AGC stock values in Alemsaga forest lies between 38.144–323.84 t/ha in the year 2022. The lowest AGC stock areas are near to farmland, grazing land, road area and less vegetation areas (38.144 t/ha to 133.38 t/ha). These areas were affected by human activities and the area covered by grass and bush or shrub lands. Dense forest areas were found the central parts of the forest, which holds AGC of 266.71–323.84 t/ha. Next to dense forest areas spares forest areas were holds 228.62–266.70 t/ha, which covered the surrounding parts of the dense forest and the bush lands of the study area. The minimum, maximum, average values & sum of AGC stock in 2022 was 38.144 t/ha, 323.84 t/ha, 228.37±57.979 t/ha and 2253300 ton, respectively (Table 5).

**Table 5:** Summary of carbon stock of Alemsaga forest from 1992–2022 in t/ha.

Year	1992	2003	2013	2022
**Minimum**	8.1077	24.644	36.771	38.144
**Maximum**	159.31	250.15	262.93	323.84
**Average**	84.581	134.58	155.27	228.37
**Standard deviation**	24.625	38.804	38.484	57.979
**Sum**	834560	1327900	1532000	2253300

Table 5 showed that the minimum, maximum, average, standard deviation, and sum of the AGC stock value of the Alemsaga forest ecosystem in 1992 were 8.1077 t/ha, 159.31 t/ha, 84.581 t/ha, 24.625 t/ha, and 834560 t/ha respectively. Whereas, in the years 2003, 2013, and 2022, the minimum, maximum, average, and sum of AGC stock values were 24.644 t/ha, 36.771 t/ha, 38.144 t/ha, 250.31 t/ha, 262.93 t/ha, 323.84 t/ha, 134.58 t/ha, 155.27 t/ha, 228.37 t/ha, 1327900 tone, 15322000 tone, and 2253300 tone, respectively.

Generally, according to Ethiopia’s Green Legacy Initiative (EGLI), forest protection and plantations have increased since the eve of the third millennium in the current status. This leads to an increase in the magnitude of AGC stock values in the Alemsaga forest ecosystem. From 1992 to 2022, 164.53 t/ha of AGC stock values increased over 30-year in Alemsaga forest. This implies that when the area protected from any external pressure rapidly increased the carbon stock values and CO_2_ sequestration over the protection area.

### Validation of the model

**Fig 12:**
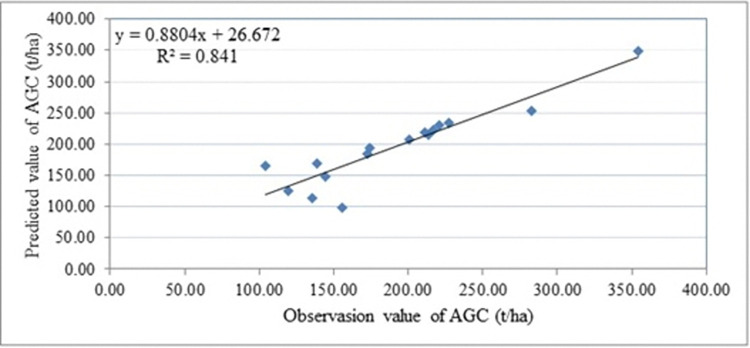
Scattered plot predicted versus observed AGC (t/ha) in Alemsaga forest.

The validation of the model checked from the collection data of 16 sample plots and predicted values by using the allometric equation. Fig 12 showed that this study developed simple linear regression model to validate the AGC stock values of 16 sample plots from 36 sample plots in Alemsaga forest.

The most straightforward and often utilized allometric formula for carbon or biomass prediction is a linear model with the formula *y* = *a*×+*b* [56]. The study employed the linear regression equation, anticipating that *a* = 1 and *b* = 0. The regression’s results were then entered into the equation as *y* = 0.880×26.672. The findings indicate that slope *a*<1, and intercept *b*>0. The R^2^ = 0.841 indicating a significant correlation between the observed and predicted values of AGCS. This suggests that 84% of the AGCS observed values were explained by the predicted values, whilst 16% of the AGCS observed values were not explained by the predicted values. From the 16 validation sample points, the RMSE of the observed and predicted values of AGCS was 17.71, with a difference between 1.56 & 61.87 t/ha.

### Conclusion and recommendations

A study in Alemsaga, Ethiopia, investigated using vegetation indices from Landsat satellites (TM, ETM+, and OLI) to estimate forest carbon. They found these indices effective with ground measurements for building models to assess carbon stock. This highlights the value of satellite data in environmental monitoring. NDVI and DVI were the two VIs that were the focus of the analysis. The findings indicate that around 79% of the variation in carbon stock across the research plots can be explained by these indexes. Other factors that are not covered by these VIs probably have an impact on the remaining 21%. NDVI and DVI had the strongest correlation with carbon stock at the plot level, despite the evaluation of other VIs as well. The study in 2022 found that carbon stock in Alemsaga forest ranged from 38 to 324 tons per hectare (t/ha). Areas with lower carbon stock (38–133 t/ha) were near farmland, grazing lands, roads, and less vegetated areas, likely impacted by human activity and dominated by grass and shrubs. Denser forest areas in the central parts held the most carbon (267–324 t/ha), followed by sparser forests (229–267 t/ha) surrounding them and shrublands. Overall, the estimated total carbon stock in the forest increased significantly from 2013 to 2022, with an average stock also rising.

Environmentalists and experts in the subject might find this research’s useful tool for calculating forest carbon interesting. Policymakers can utilize the findings to help set reasonable development targets, make well-informed decisions about land use, and increase carbon storage to promote a more balanced global climate. Still, greater investigation of other data sources such as Lidar or Pol-InSAR is necessary to get even more precise estimates of the future carbon content of this forest region.
